# Physiological and proteomic analyses of leaves from the halophyte *Tangut Nitraria* reveals diverse response pathways critical for high salinity tolerance

**DOI:** 10.3389/fpls.2015.00030

**Published:** 2015-02-10

**Authors:** Tielong Cheng, Jinhui Chen, Jingbo Zhang, Shengqing Shi, Yanwei Zhou, Lu Lu, Pengkai Wang, Zeping Jiang, Jinchang Yang, Shougong Zhang, Jisen Shi

**Affiliations:** ^1^Key Laboratory of Forest Genetics and Biotechnology, Ministry of Education, Nanjing Forestry UniversityNanjing, China; ^2^State Key Laboratory of Tree Genetics and Breeding, Chinese Academy of ForestryBeijing, China; ^3^Experimental Center of Desert Forestry, Chinese Academy of ForestryBeijing, China; ^4^Research Institute of Tropical Forestry, Chinese Academy of ForestryGuangzhou, China

**Keywords:** iTRAQ, halophyte, responsive pathways, salinity tolerance, *Tangut Nitraria*

## Abstract

Soil salinization poses a serious threat to the environment and agricultural productivity worldwide. Studies on the physiological and molecular mechanisms of salinity tolerance in halophytic plants provide valuable information to enhance their salt tolerance. *Tangut Nitraria* is a widely distributed halophyte in saline–alkali soil in the northern areas of China. In this study, we used a proteomic approach to investigate the molecular pathways of the high salt tolerance of *T. Nitraria*. We analyzed the changes in biomass, photosynthesis, and redox-related enzyme activities in *T. Nitraria* leaves from plant seedlings treated with high salt concentration. Comparative proteomic analysis of the leaves revealed that the expression of 71 proteins was significantly altered after salinity treatments of *T. Nitraria*. These salinity-responsive proteins were mainly involved in photosynthesis, redox homeostasis, stress/defense, carbohydrate and energy metabolism, protein metabolism, signal transduction, and membrane transport. Results showed that the reduction of photosynthesis under salt stress was attributed to the down-regulation of the enzymes and proteins involved in the light reaction and Calvin cycle. Protein–protein interaction analysis revealed that the proteins involved in redox homeostasis, photosynthesis, and energy metabolism constructed two types of response networks to high salt stress. *T. Nitraria* plants developed diverse mechanisms for scavenging reactive oxygen species (ROS) in their leaves to cope with stress induced by high salinity. This study provides important information regarding the salt tolerance of the halophyte *T. Nitraria*.

## Introduction

Salinization is one of the world's most serious environmental factors that affect the growing area and productivity of many plants (Stepien and Johnson, 2009). More than 6% of the world's land is affected by high salinity (Munns and Tester, 2008), and increased salinization may lead to a 30% loss of arable land in the next 25 years and up to 50% by 2050 (Wang et al., 2008). The increasing damage caused by high salinity has prompted extensive research on plant responses and adaptation mechanisms (Zhu, 2001; Munns and Tester, 2008).

Plants exhibit evolved complex mechanisms, including selective ion uptake/exclusion, adjustment of photosynthesis and energy metabolism, accumulation of antioxidative enzymes, and synthesis of substances, to cope with salinity stress (Zhang et al., 2011). Plants are categorized based on their responses to salinity into halophytes and glycophytes. Halophytes are innate to environments with high degrees of salinity, whereas glycophytes cannot tolerate similar salt levels (Chen et al., 2012). Compared with glycophytes, halophytes have developed unique structures, including salt glands, bladder hairs, succulent tissues, and thick layers of suberin, to tolerate salt stress (Flowers and Colmer, 2008).

Transcriptional analyses on halophytes, including *Thellungiella halophila* (Wong et al., 2006), *Aeluropus littoralis* (Zouari et al., 2007), *Salicornia brachiata* (Jha et al., 2009), and *Festuca rubra* ssp. *litoralis* (Diédhiou et al., 2009), have been performed under salt stress to elucidate their salt-tolerance mechanisms. Compared with plants exposed to high salt concentrations, halophytes treated without salt stress differentially express ESTs/cDNAs. These proteins are mainly involved in signaling, photosynthesis, transcriptional regulation, reactive oxygen species (ROS) scavenging, and ion homeostasis. Studies at the protein level have used comparative proteomic approaches to investigate salt-tolerance mechanisms in halophytes, including *Salicornia europaea* (Wang et al., 2009), *Suaeda aegyptiaca* (Askari et al., 2006), *Bruguiera gymnorhiza* (Tada and Kashimura, 2009), and *T. halophila* (Pang et al., 2010). Halophytes contain more salt-responsive genes than glycophytes. Some salt-responsive genes from halophytes have been cloned and transferred into glycophytes to improve their salt tolerance (Flowers and Colmer, 2008). Complex and sophisticated molecular networks with common and specific characteristics can control salt tolerance in plants (Yu et al., 2011; Zhang et al., 2011).

*Tangut Nitraria*, a dicotyledonous halophyte that belongs to the family Zygophyllaceae *Nitraria*, exhibits high salt tolerance (Chen et al., 2012). This plant is common in Inner Mongolia, Gansu, Qinghai, Xinjiang, and other regions of the Gobi desert of China. It is important in maintaining an ecologically balanced vegetation in these regions. *T. Nitraria* prevents desertification by fixing sand, improving soil, and maintaining an ecological balance within the sandy area (Chen et al., 2012). Although *T. Nitraria* can readily adapt to high salt conditions, little is known about the molecular mechanisms and regulatory networks involved.

To elucidate the molecular mechanisms involved in the response of the genus *Nitraria* to high salinity, we previously analyzed the dynamic protein expression patterns in *Nitraria sphaerocarpa* cell suspensions under salinity stress (Chen et al., 2012). Halophytes exhibit high tolerance because of the specific mechanisms for salt exclusion by the roots, vascular compartmentation of tissue solutes, and leaf excretion of excess salt; each tissue plays different and specific roles in response to high salt stress. In this study, we exposed *T. Nitraria* to 500 mM NaCl for 1, 3, 5, and 7 days and then analyzed the changes in the physiology and expression of salt-responsive proteins in the leaves using isobaric tags for relative and absolute quantitation (iTRAQ) approach. Bioinformatics analysis comprehensively revealed the linkage between protein abundance changes and diverse metabolic pathways affected by high salinity.

## Materials and methods

### Growing conditions and salt treatment of *T. Nitraria*

*T. Nitraria* seeds were embedded in plastic pots (14 cm high, 12 cm diameter, with holes at the bottom) filled with washed river sand. Five pots were placed in a plastic tub (15 cm high and 80 cm diameter). The seedlings were transferred to tubs filled with tap water and then placed in a greenhouse under 14 h of light (400–800 μmol m^−2^ s^−1^) at 27±2°C and 10 h of darkness at 25±1°C. Relative humidity was maintained at 60–80%. Two-month-old healthy seedlings were irrigated with half-strength Hoagland's nutrient solution. The seedlings with uniform sizes were divided into five groups and then treated with 500 mM NaCl for 1, 3, 5, and 7 days. The leaves of each seedling were harvested after 0, 1, 3, 5, and 7 days of treatment for further analysis. At least three independent replicates were conducted in each treatment for all experiments.

### Measurement of leaf biomass and ultrastructure

Fresh weight (FW) of *T. Nitraria* leaves was immediately obtained after treatment. Dry weight (DW) was determined after dehydration at 90°C until a constant weight was reached. Leaf water content was estimated as the difference of the FW and DW divided by the FW (Askari et al., 2006). Leaf ultrastructure was analyzed using the method described by Bai et al. (2011). Images were obtained using a transmission electron microscope (TEM-100CX II, Japan).

### Relative electrolyte leakage assay and photosynthesis measurement

Relative electrolyte leakage was measured using the method described by Yan et al. (2006) with the following modifications. Leaves were cut into 0.5 cm segments and washed three times with ultrapure water. Each segment was placed in a tube containing 10 ml of ultrapure water and then incubated at 28°C. After 2 h, the electrical conductivity of the bathing solution (*L_t_*) was measured using a conductometer (DDSJ-308A, China). The tubes were incubated at 100°C for 30 min and 28°C for 1 h before measuring the electrical conductivity (*L*_0_). The relative electrolyte leakage was calculated as *L_t_*/*L*_0_ × 100%. Five replicates were performed for each sample.

The net photosynthetic rate (Pn), stomatal conductance (Gs), intercellular CO_2_ (Ci), and transpiration rate (Tr) were determined using the portable photosynthesis system LICOR 6400 (LI-COR Inc., USA). Eight leaves were measured for each sample in each experiment. Experiments were performed three times. Chlorophyll fluorescence (*Fv/Fm*) was measured in random, fully expanded leaves using the portable photosynthesis system LICOR 6400 (LI-COR Inc., USA) at mid-day on each plant of salinity treatment. Measurements were made on six plants from each of the salinity treatments according to the LICOR 6400 user manual and the method of Redondo-Gómez et al. (2007).

Lipid peroxidation was determined by measuring the malondialdehyde (MDA) content (Dhindsa and Matowe, 1981). In brief, leaves (0.5 g) were homogenized with 5 ml of trichloroacetic acid (0.1%, v/v) and then centrifuged at 15,000 *g* for 30 min. The supernatant was collected and mixed with 20% (v/v) trichloroacetic acid and 0.5% (v/v) thiobarbituric acid. The mixture was heated at 95°C for 30 min, rapidly cooled, and centrifuged at 15,000 *g* for 30 min. The MDA concentration in the supernatant was detected at 532 nm.

### Determination of potassium and sodium

Leaf samples were dried for 30 min at 105°C, and then dried at 80°C for about 48 h until a constant weight was maintained. Then, dried leaves were ashed at 500°C and extracted with HNO_3_. Potassium and sodium concentrations in the leaves were measured using an atomic absorption spectrophotometer (AA240; Varian Medical Systems, Palo Alto, CA, USA) according to the method reported by Munns et al. (2010).

### Antioxidative enzyme activity assay

Enzyme activity assays of superoxide dismutase (SOD), catalase (CAT), and peroxidase (POD) were conducted using the methods reported by Yang et al. (2013) with the following modifications. In brief, leaves (1 g) were homogenized on an ice bath in 5 ml of buffer I containing 50 mM sodium phosphate buffer (pH 7.8), 0.1 mM ethylenediaminetetraacetic acid, 4% polyvinylpolypyrrolidone, and 0.3% (v/v) Triton X-100. After centrifugation at 15,000 g and 4°C for 20 min, enzyme activity was detected in the supernatants.

### Protein extraction and quantification

Protein extraction and quantification were conducted using the methods reported by Chen et al. (2012) with the following modifications. The leaves (5 g) of the plants treated with 500 mM NaCl for 0, 1, 3, 5, and 7 days were collected and pulverized in a mortar grinder with liquid nitrogen. Lysis buffer (2 M thiourea, 7 M urea, and 4% CHAPS, pH 8.5) was added to the powder, and the mixture was thoroughly vortexed. The mixture was then centrifuged at 40,000 g for 30 min, and the pellet was discarded. Proteins were precipitated with acetone containing 10% (w/v) trichloroacetic acid at −20°C to remove plant pigments. The precipitated proteins were collected by centrifugation at 40,000 g for 30 min, and washed with acetone two to three times until the pellet became colorless. The pellets were dried under vacuum and then dissolved in 8 M urea supplemented with 10 mM DTT (pH 8.5). The protein concentration was determined using Bradford assay.

### Trypsin digestion and iTRAQ labeling of *T. Nitraria* proteins

Reagents and buffers used for iTRAQ labeling and cleaning were obtained from Applied Biosystems (Foster City, CA, USA). iTRAQ was performed following the manufacturer's instructions. In brief, the proteins were dissolved, denatured, alkylated, and digested with trypsin (Sigma) at 37°C for 18 h. To label peptides with iTRAQ reagent, one unit of label (defined as the amount of reagent required to label 100 g of protein) was thawed and reconstituted in 70 μl of ethanol. Prior to digestion, the control samples (0 h) were labeled with 113 iTRAQ reagent, whereas the saline-treated leaves (1, 3, 5, and 7 h) were labeled with 114, 115, 116, and 117 iTRAQ reagents, respectively. The mixed peptides were separated using a strong cation exchange column (SCX, 0.75 × 20 mm, Applied Biosystems) and Thermo BioBasic SCX column. The following elution buffers were used: elution buffer A containing 5 mM K_2_HPO_4_ in 20% (v/v) acetonitrile (pH 3.0) and elution buffer B consisting of 5 mM K_2_HPO_4_ in 20% (v/v) acetonitrile and 350 mM KCl (pH 3.0). The labeled peptides were diluted in buffer A and injected at a flow rate of 0.7 ml/min onto a high-resolution SCX column (4.6 × 250 mm, 5 μm; Thermo BioBasic, USA). After loading, the SCX column and C18 pre-column were flushed with a three-step gradient NaCl solution (0, 50, and 100 mM) for 66 min, and the pH of the diluted sample was adjusted between 2.5 and 3.3. The diluted sample mixture was loaded onto the cation exchange cartridge and then washed with 10 column volumes of buffer load. The peptides were eluted with 500 μl of buffer elute (10 mM K_2_HPO_4_ in 25% (v/v) acetonitrile and 350 mM KCl, pH 3.0). Subsequently, the eluate of the cation exchange column was desalted with an Agilent 1100 series HPLC system equipped with an auto sampler, 2/6 valve, and diode array detector (220 nm) (Agilent, Waldbronn, Germany). Thirty fractions were collected.

### Peptide analysis via triple quadrupole time-of-flight (TOF) tandem mass spectrometry (MS)

The fractionated peptides were analyzed using a triple quadrupole TOF 5600 system (AB SCIEX, Concord, ON) fitted with a Nanospray III source (AB SCIEX, Concord, ON, USA) and pulled quartz tip as the emitter (New Objectives, Woburn, MA, USA). Data were acquired using an ion spray voltage of 2.5 kV, curtain gas of 30 PSI, nebulizer gas of 6 PSI, and interface heater temperature of 150°C. The mass spectrometer was operated with an RP of 30,000 FWHM for TOF-MS scans. For information-dependent acquisition, survey scans were acquired at 250 ms. A total of 20 product ion scans were collected, and ions exhibited a charged state of +2 to +5 if 125 counts per second were exceeded. A rolling collision energy setting was applied to all precursor ions for collision-induced dissociation.

### Construction and search of *T. Nitraria* polypeptide/protein database

Transcriptome sequencing and assembly of leaves from *T. Nitraria* seedlings were conducted using our method (Wang et al., 2013). The assembled unigenes were used to construct conceptual protein/peptide sequences following our reported procedure (Chen et al., 2011). By comparing the BlastP results against the NCBInr protein database, all possible polypeptide sequences were extracted and annotated to develop a local protein/peptide sequence database of *T. Nitraria*. All sequences were converted into FASTA format for further application.

ProteinPilot software 4.0 (AB SCIEX, Foster City, CA, USA), including the Paragon and Pro Group™ algorithms, was used to identify proteins and interpret raw data from MS analysis (Yang et al., 2014). The peptides and corresponding relative abundances were obtained with ProteinPilot using a confidence cutoff (called a “Prot Score”) of >1.0 (>90%) (Martínez-Esteso et al., 2013). The parameters for database searching were as follows: the peptide should be iTRAQ-labeled, trypsin digestion with only one missed cleavage, carboxymate formation for cysteine residues, oxidation for methionine, and instrument set as qTOF-ESI. Tolerance was specified as ±0.05 Da for peptides and MS/MS fragments. Local protein/peptide database of *T. Nitraria* was queried for protein identification. Only the proteins identified with at least two different peptides and *p* < 0.05, and quantified with a ratio of >1.5 and *p* < 0.05, were considered. The former *p*-value was related to the protein score cutoff in the identification, whereas the latter *p*-value was related to the iTRAQ ratio for each quantified protein and computed from the ProGroup Algorithm in ProteinPilot software as a measure of statistical significance (Martínez-Esteso et al., 2013). The protein/peptide database of *T. Nitraria* was constructed using the transcript data from our laboratory (Chen et al., 2011). FDR was controlled at 1% using the integrated tools in ProteinPilot. For protein assembly, the Pro Group algorithm was used to determine the smallest number of proteins that could explain protein fragmentation patterns.

Protein quantification was also performed using ProteinPilot software, which automatically calculated the relative abundance of iTRAQ-labeled peptides and corresponding proteins. Corrections were applied for impurities of iTRAQ reagents based on the data provided by the manufacturer. For other similar analysis errors, iTRAQ ratios were normalized using the auto-bias function.

### Functional annotation and classification of identified proteins

The functions of differentially expressed proteins were assigned using the protein function database Pfam (http://www.sanger.ac.uk/Software/Pfam/) (Finn et al., 2008) or InterPro database (http://www.ebi.ac.uk/interpro/) (Apweiler et al., 2001). Annotation and categorization were performed similar to the methods used for *Arabidopsis* (Bevan et al., 1998). The molecular functions and biological processes of the identified proteins were classified using DAVID program (http://david.abcc.ncifcrf.gov) for Gene Ontology (GO) and KEGG annotation. The protein–protein interaction network was constructed using the String program. Moreover, we used the data from genomic prediction models, results from high-throughput experiments, and previous knowledge regarding co-expression in plants to construct our interaction network.

### Statistical analysis

All data were subjected to ANOVA using SPSS software version 6.0, and means (*n* = 5) were separated using the Fisher's least-significant difference test at >95% confidence interval (*p* < 0.05).

## Results

### Effect of high salinity on the morphology and thylakoid ultrastructure of *T. Nitraria* seedling leaves

**Figure 1:**
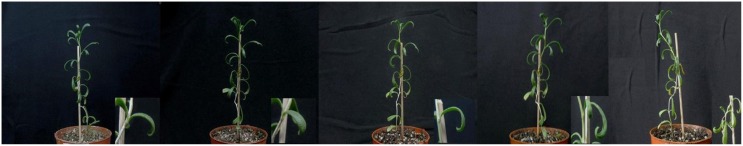
**Morphological changes in *T. Nitraria* seedlings after treatment with 500 mM NaCl for 0, 1, 3, 5, and 7 days**. The photographs show similar plants at the indicated time points. The insets in the lower right corner depict an enlarged region of the plant. The photographs shown are representative of five independent experiments.

**Figure 2:**
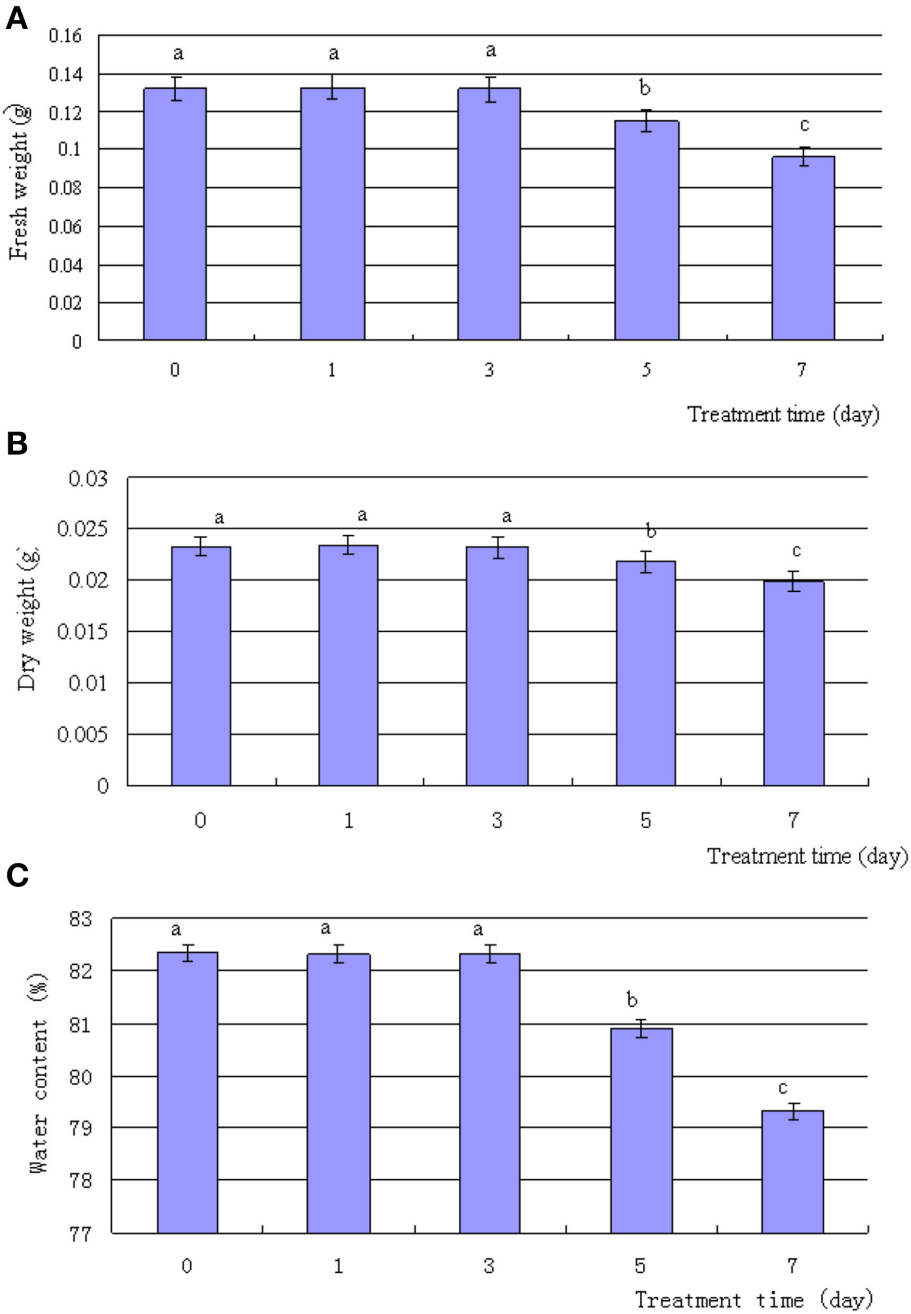
**Effects of salinity stress on the weight and water content of *T. Nitraria* leaves**. *T. Nitraria* seedlings were treated with 500 mM NaCl for 0 (control), 1, 3, 5, and 7 days. The changes in fresh weight, dry weight, and water content of the leaves are also shown (**A**, **B**, and **C**, respectively). The values are presented as means ± standard error (SE); *n* = 6 for all groups. The bars represent the SE. Bars with the same letter are not significantly different (*P* < 0.05).

The exposure of *T. Nitraria* seedlings to 500 mM NaCl resulted in various morphological and physiological changes in the leaves over time. Although treatment with 500 mM NaCl for up to 3 days did not induce any evident phenotypic differences in the seedling leaves, treatment for 5–7 days induced the leaf margins to roll inward (Figure 1). After 5 days of treatment, the leaves of salt-treated seedlings started to wilt and displayed severe symptoms, such as curling at day 7 (Figure 1). The FW and DW (Figures 2A,B), as well as the water content (Figure 2C), of the leaf decreased after 5 and 7 days of treatment, with no significant reduction during the first 3 days of treatment. Thus, high salinity treatment for a long time period evidently suppressed the normal growth of *T. Nitraria* seedlings and reduced the FW and DW of their leaves.

**Figure 3:**
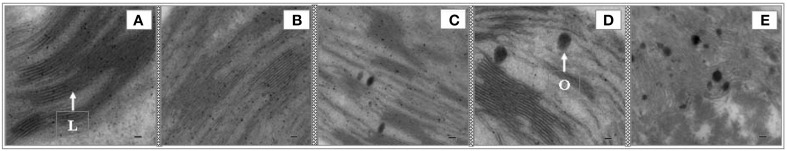
**Effects of salinity on the chloroplasts of *T. Nitraria* seedling leaves after treatment with 500 mM NaCl**. Images of the leaves were obtained using a transmission electron microscope (TEM-100CX II, Japan). **(A)** Control (0 day); **(B)** 1 day; **(C)** 3 days; **(D)** 5 days; and **(E)** 7 days of treatment. The scale bar is equal to 2.0 μm. L, stroma lamellae; O, osmiophilic body.

To evaluate the ultrastructural changes induced by high salinity in the chloroplast, we prepared the leaves from the control and NaCl-treated seedlings for transmission electron microscopy. Compared with the control, the structure of thylakoids in the chloroplasts of leaf mesophyll cells changed shape during high salinity treatment for 7 days (Figure 3), but the chloroplasts in the mesophyll cells appeared similar. Priorto salt treatment and during 1–3 days of treatment, no evident differences were detected in the thylakoid structure (Figures 3A–C). At day 5, thylakoids showed changes in shape, involving ambiguity of the membrane, loose grana lamella, and thickening on the edges of grana lamella. At day 7, the thylakoid membranes disintegrated, thylakoids were irregularly arranged, and stroma lamellae swelled and cracked. The increased number and enlarged volume of osmiophilic bodies (Figures 3C–E) showed that high salinity treatments disrupted the granum thylakoid and possibly affected the function of chloroplasts.

### Photosynthetic responses and changes of chlorophyll fluorescence induced by salinity stress in *T. Nitraria* seedling leaves

**Figure 4:**
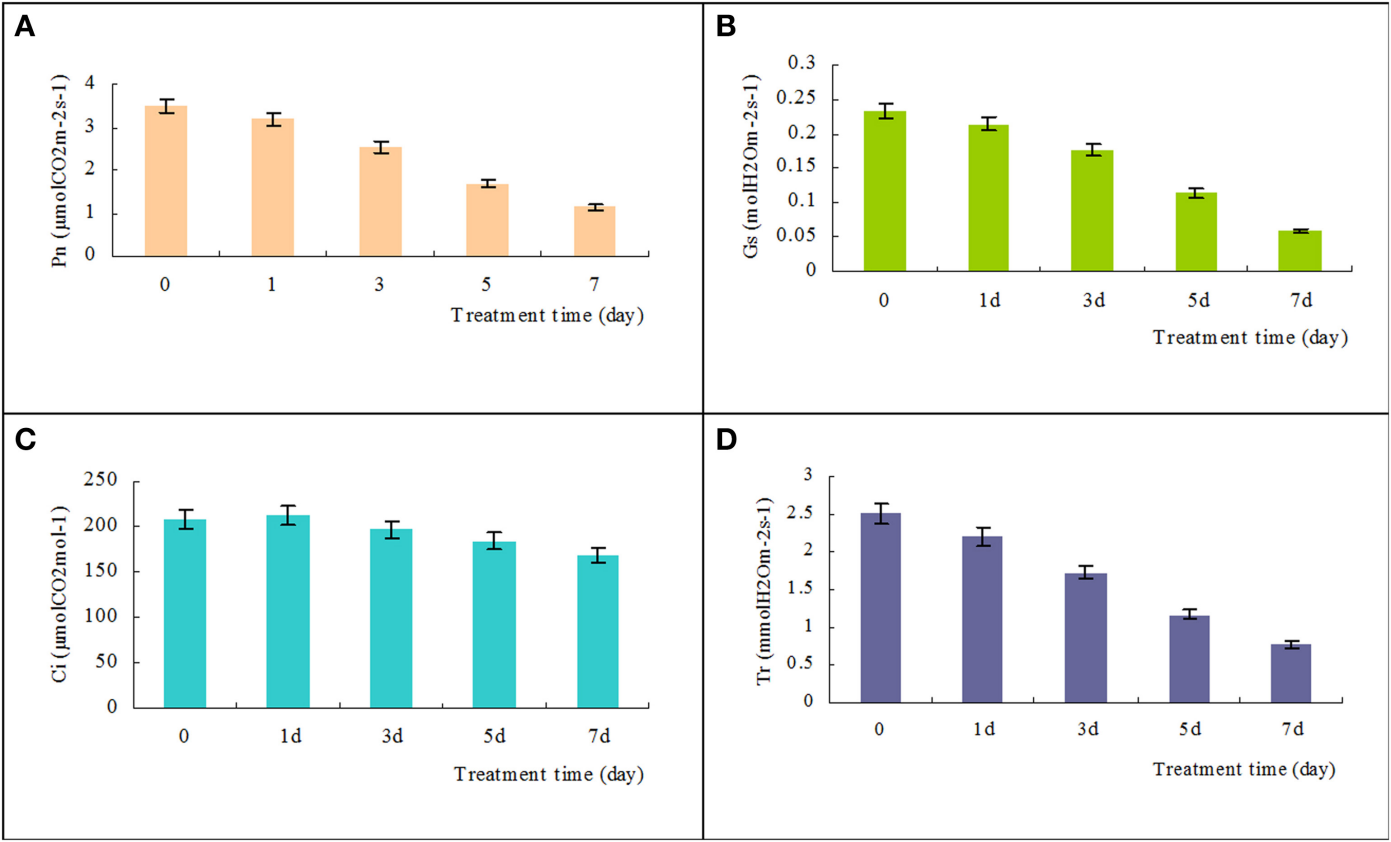
**Effects of high salinity on the net photosynthetic rate (Pn), stomatal conductance (Gs), intercellular CO_2_ (Ci), and transpiration rate (Tr)**. Changes in Pn, Gs, Ci, and Tr in *T. Nitraria* seedling leaves after treatment with 500 mM NaCl for 0, 1, 3, 5, and 7 days were determined using the portable photosynthesis system LICOR 6400 (LI-COR Inc., USA) (**A**, **B**, **C**, and **D**, respectively). The values are presented as means ± standard error (SE); *n* = 6 for all groups. The bars represent the SE. Bars with the same letter are not significantly different (*P* < 0.05).

To evaluate the adverse effects of salinity stress, we measured the photosynthetic responses of *T. Nitraria* seedling leaves after saline stress treatments. The Pn significantly decreased from the initial value of 3.5 μmol CO_2_ m^−2^s^−1^ to 2.5, 1.7, and 1.1 μmol CO_2_ m^−2^ s^−1^ after 3, 5, and 7 days of treatment, respectively (Figure 4A). The Gs and Tr also decreased (Figures 4B,D), thereby indicating that salinity treatment resulted in stomatal closure, reduced water transpiration, and reduced photosynthesis in *T. Nitraria* seedling leaves. By contrast, no evident changes were observed in the intercellular CO_2_ (Ci) levels in response to salinity stress (Figure 4C). Salinity treatment mitigated the inhibitory effect on the photosynthetic capability of *T. Nitraria* seedling leaves. Similar results were observed in saline-treated mangrove *Kandelia candel Druce* seedlings (Wang et al., 2013), which suggested that salinity stress is related to photosynthesis inhibition in plants (Zhang et al., 2011). In addition, the maximum quantum efficiency of PSII photochemistry (*Fv/Fm*) values were significantly increased in leaves of plants at 1, 3, 5, and 7 days of treatment comparing the control (Figure S1).

### Potassium and sodium concentrations in leaves under salinity stress

Sodium concentration in leaves of plants treated with high salinity for 1, 3, 5 and 7 days increased by 3.97-, 5.22-, 6.45- and 7.72-fold, respectively, compared with controls. The Na concentration in leaves of plants continually increased during the process of salinity treatment (Figure S2A). Conversely, the potassium concentration in the leaves continually decreased (Figure S2B). The K^+^/Na^+^ ratio showed a decreased pattern during the salinity treatments (Figure S2C).

### Effect of salt treatment on the activity of antioxidant enzymes and membrane status

The activities of various antioxidant enzymes varied under high salt conditions. The activities of SOD and POD initially increased during days 1–5 and then decreased at day 7 of treatment. In addition, CAT activity slightly decreased during the first three treatment days and then increased after 5 days of treatment (Figure S3). Hence, plants under saline stress may up-regulate ROS-scavenging enzyme activities to eliminate excessive oxidative stress induced by high salt concentrations.

High salt concentrations also increased electrolyte leakage from the leaves, demonstrating the extensive membrane damage and formation of membrane lipid peroxidation products, including MDA (Figure S4). The increased membrane permeability and MDA products showed that salt stress increased leaf membrane damage.

### Changes in the leaf proteome of *T. Nitraria* in response to salinity stress

Seedlings of *T. Nitraria* were subjected to 500 mM NaCl for 1, 3, 5, and 7 days, and the proteomic changes induced by this salt concentration were analyzed using iTRAQ (Figure S5). Data from three technical replicates were analyzed to detect proteins by querying the local protein/peptide database. For each replicate, the peptides were assembled into proteins. A total number of 746 proteins were produced, resulting in the identification of 502 proteins common to the three data sets with at least two unique peptides (Tables S4, S5). Statistical analysis of the proteins at various time points revealed that 71 proteins were differentially expressed (*p* = 0.05). Most of these proteins exhibited a higher than 2.0-fold change in abundance in at least one time point of salinity stress treatment (Table S1).

The 71 proteins were classified into four groups on the basis of hierarchical clustering of their relative expression abundance (Figure S6). The abundance of the proteins in the first group increased after exposure to high salt (Figure S6A), whereas that of the proteins in the second group decreased (Figure S6B). The abundance of the proteins in the third group initially decreased (days 1–3) and then increased (days 5–7) (Figure S6C), whereas that of the proteins in the fourth group initially increased (days 1–3) and then decreased at days 5–7 (Figure S6D). The increase in abundances may represent specific sensitivity or adaptation of plants to salinity stress, and the decrease in abundance may reflect the cellular damage caused by exposure to salinity conditions. Our observations suggested that *T. Nitraria* plants monitored the extent of salinity stress and alleviated salinity-induced damage by modulating protein expression.

### Identification and functional categorization of differentially expressed proteins

To identify differentially expressed proteins, we searched the NCBInr protein database for homologous sequences using BLASTP (www.ncbi.nlm.nih.gov/BLAST/). The proteins identified were displayed using proteins with highest sequence similarities (Table S1). Protein sequences with >60% homology were assumed to exhibit similar functions, and found in the Pfam or Inter-Pro database. Two proteins (Unigene46915_1_1 and Unigene39818_1_1) were annotated as unknown or hypothetical proteins (Table S1).

The differentially expressed proteins were sorted into 13 functional categories, namely, photosynthesis, redox homeostasis, stress and defense, energy metabolism, carbohydrate metabolism, amino acid metabolism, signal transduction, protein synthesis, protein folding and assembly, transcription, membrane and transport, hormone synthesis, and others or unknown (Figure S7). The most represented proteins (the number of proteins falling into a specific category) were associated with carbohydrate metabolism (20%), redox homeostasis (17%), and photosynthesis (13%). The proteins in these first three categories were overrepresented, suggesting that they were important for salinity stress resistance and adaptation. Similar results were observed by Yu et al. (2011), who analyzed salinity-induced changes in the seedling proteome of the halophyte *Puccinellia tenuiflora*.

### GO analysis of differentially expressed proteins

**Figure 5:**
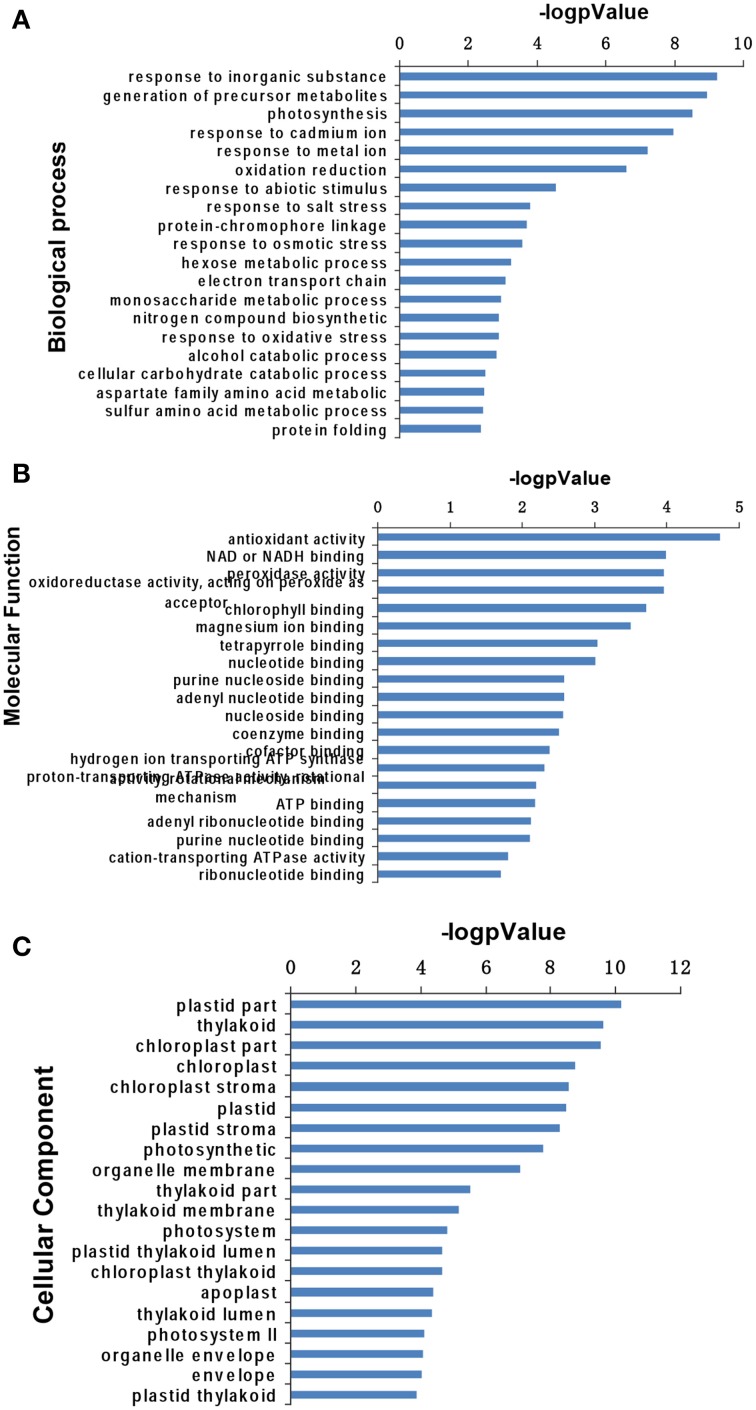
**Gene Ontology analysis of salinity stress-responsive proteins in the leaves of *T. Nitraria* seedlings**. Expressed proteins involved in biological process **(A)**, molecular function **(B)**, and cellular component **(C)** against the GO database.

To obtain further knowledge on the biological functions of salinity-responsive proteins in *T. Nitraria* seedling leaves, we separately analyzed the differentially expressed proteins against the GO database with three sets of ontologies: biological process (GO-BP), molecular function (GO-MF), and cellular component (GO-CC). The most prominent GO-BP categories were as follows: (1) response to inorganic substance, generation of precursor metabolites and photosynthesis, which exhibit important functions for salinity stress responses; and (2) response to cadmium ion, response to metal ion, oxidation reduction, response to abiotic stimulus and response to salt stress (Figure 5A, Table S2). For the GO-MF category, antioxidant activity, were the most prominent, followed by NAD or NADH binding, peroxidase activity, oxidoreductase activity, and chlorophyll binding (Figure 5B, Table S2). For the GO-CC category, plastid part, thylakoid and chloroplast were the top three categories (Figure 5C, Table S2). These GO analysis results showed that salinity stress significantly affected stress defense, cellular homeostasis, and metabolism-related pathways.

### Protein–protein interaction analysis

**Figure 6:**
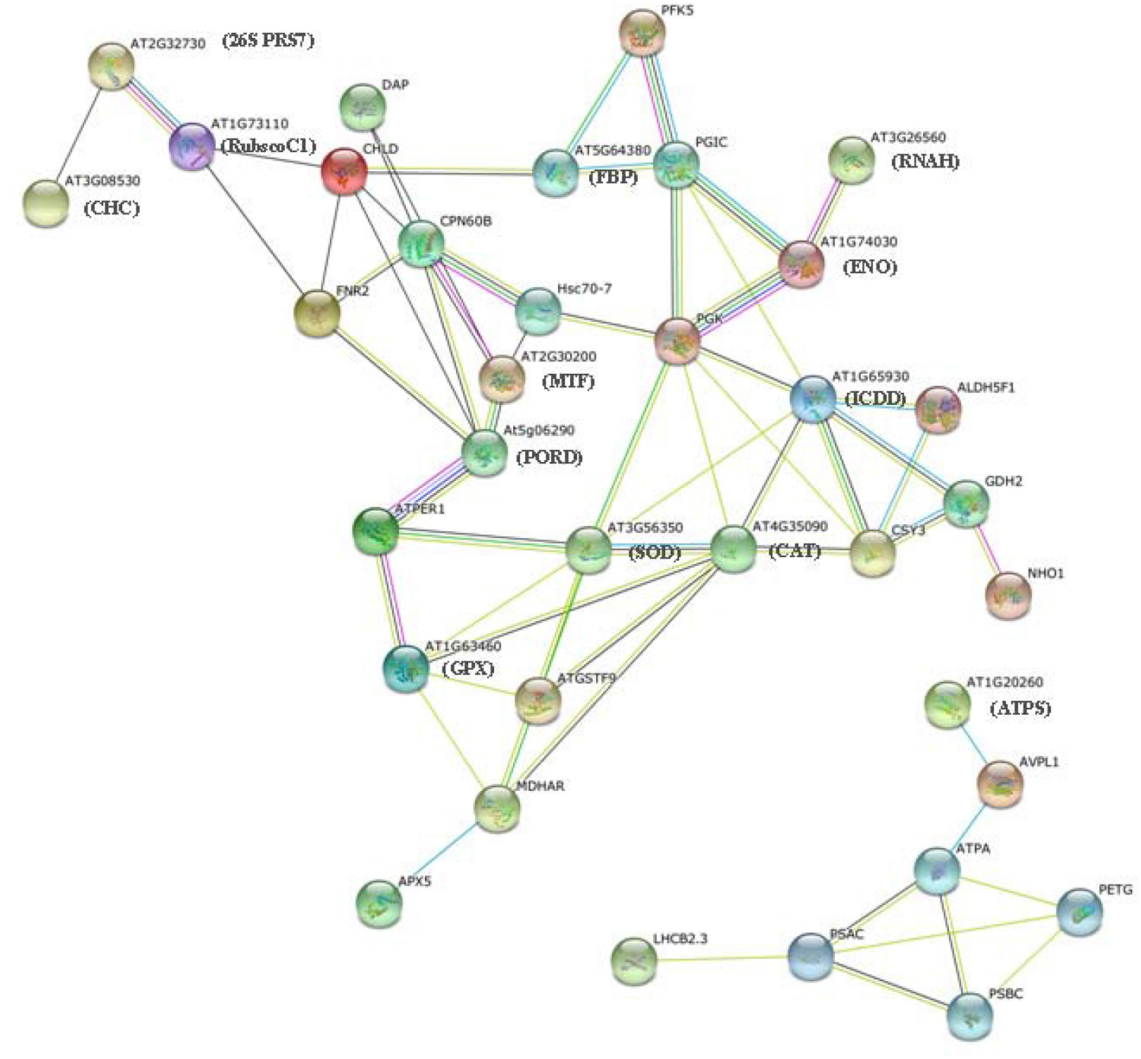
**Protein–protein interaction network analysis of salinity stress-responsive proteins**. The data categorized in A and B show two independent groups of interaction networks.

Proteins in a living cell do not function as single entities but work together in networks. We aimed to determine the mechanism underlying the transmission of salinity stress signals through protein–protein interactions in plants and effects on cell functions in *T. Nitraria* leaves. We searched for proteins that significantly changed in abundance against the String database and protein–protein interaction network. The resulting networks were constructed using String software with Confidence Scores greater than 0.7 (Figure 6) (Franceschini et al., 2013). The abbreviated names of specific proteins in the networks are shown in Supplemental Table S3. Two groups of protein interactions were detected. The proteins in the first group involved in stress resistance, antioxidant and redox homeostasis, amino acid metabolism, carbohydrate metabolism, protein metabolism, and kinase activity (Figure 6, Table S3). These results showed that the proteins in this network played important functions in redox homeostasis, response to stress, signal transduction, and carbohydrate and protein metabolism. Protein interactions in the second group mainly involved in photosynthesis and energy metabolism (Figure 6, Table S3).

In addition, the connectivity of the proteins in this biological network can provide insight into their relative homeostasis and importance in cellular activities. Protein “hubs” (connected to many other proteins) and “bottlenecks” (key connectors of sub-networks within a network), such as ATPER1 (thioredoxin peroxidase) and PGK (phosphoglycerate kinase), represent the central points for controlling communication within a network and play essential roles in the leaf response to salinity.

## Discussion

### High salt concentration targets photosynthesis

**Figure 7:**
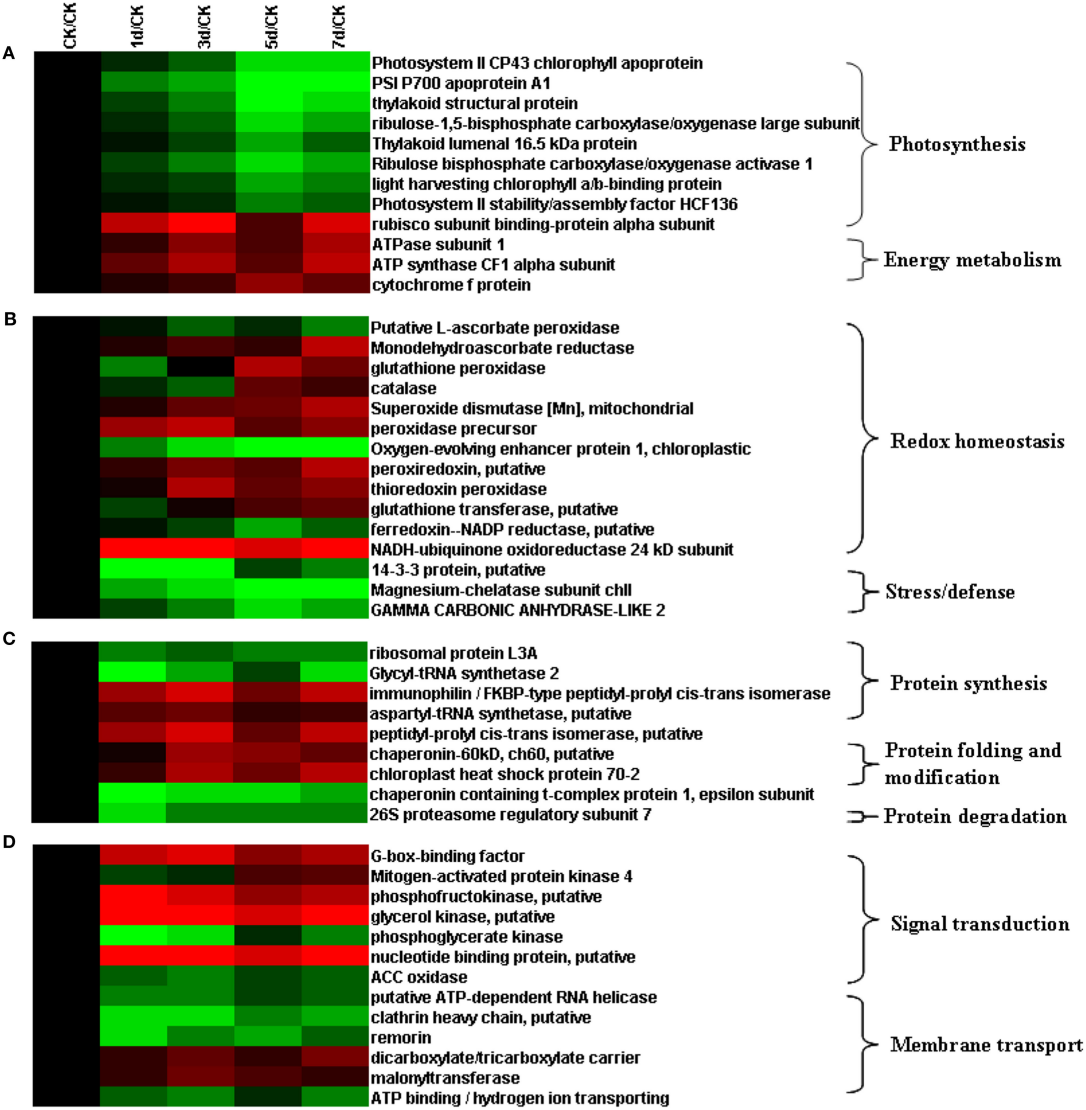
**Hierarchical clustering of salinity stress-responsive proteins involved in (A) photosynthesis, (B) antioxidative reactions, (C) protein metabolism, and (D) signal transduction**.

High degree of salinity perturbs water uptake and biosynthesis of abscisic acid in plant leaves, resulting in rapid alterations in stomatal conductance and affecting photosynthesis (Fricke et al., 2004). Similarly, exogenous high salinity treatment impaired the photosynthetic function of *T. Nitraria* seedlings in the present study, such as the decreases of Pn, Gs, and Tr (Figure 4). We identified nine differentially expressed proteins that belonged to distinct identity categories associated with photosynthesis. These proteins included PSI P700 apoprotein A1, photosystem II CP43 chlorophyll apoprotein, photosystem II stability/assembly factor HCF136, light-harvesting chlorophyll a/b-binding protein, thylakoid structural protein, ribulose-1,5-bisphosphate carboxylase/oxygenase large subunit, ribulosebisphosphate carboxylase/oxygenaseactivase 1, rubisco subunit binding-protein alpha subunit, and thylakoid lumenal 16.5 kDa protein, which showed the decrease trends under salinity stress (Figure 7A). These proteins play a role in the light reactions and Calvin cycle (Chaves et al., 2009).

**Figure 8:**
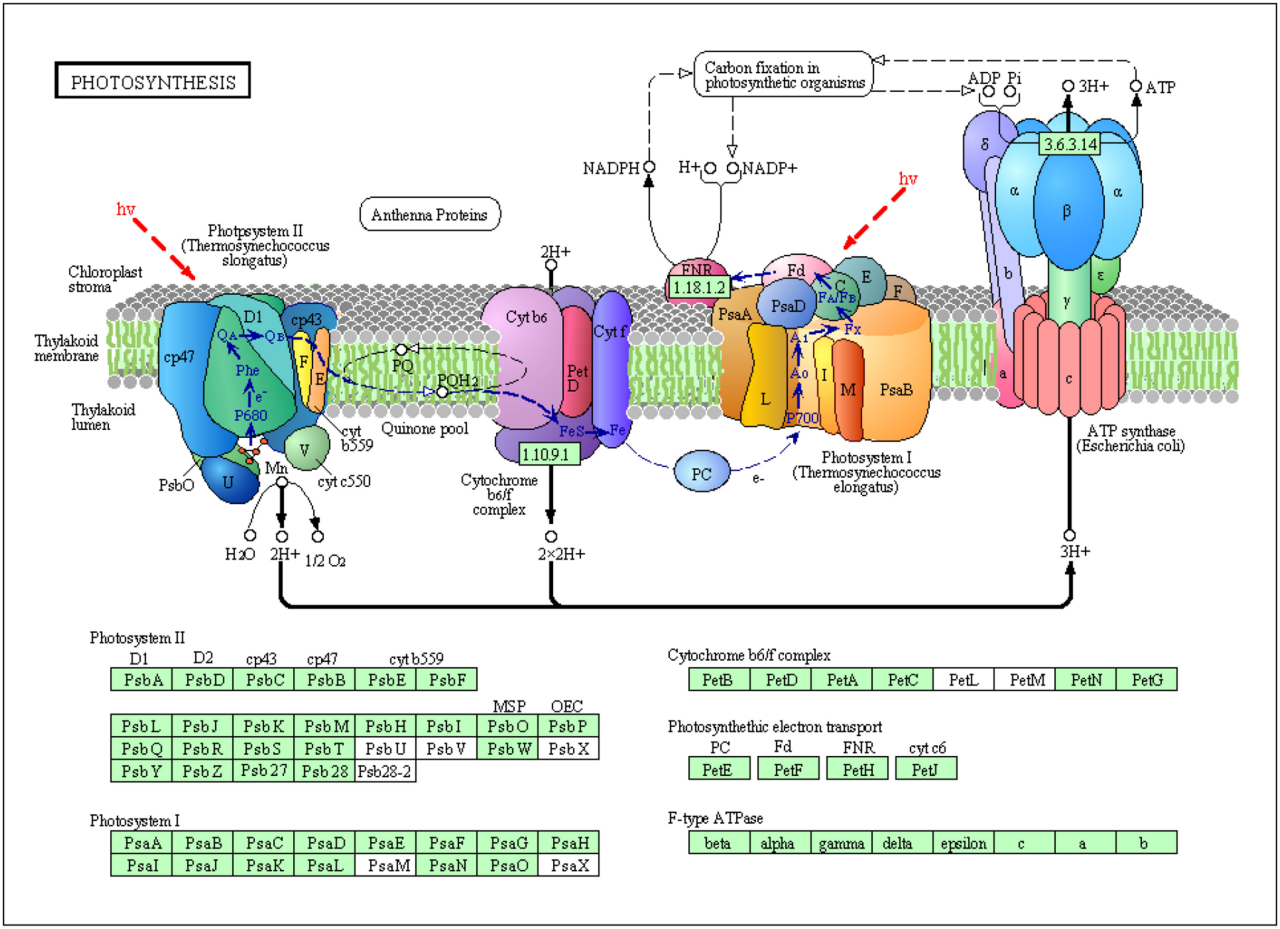
**Schematic presentation of salinity stress-responsive proteins involved in photosystem and electron transport in *T. Nitraria* seedlings**.

Two major protein complexes conduct light-dependent reactions: PSI (cytochrome complex) and PS II; ATP synthase comprises the first stage of photosynthesis, in which light is converted into chemical energy (Figure 8) (Wang et al., 2013). In the present study, 12 proteins required for light reactions were differentially expressed. Among these proteins, photosystem II CP43 chlorophyll apoprotein, light-harvesting chlorophyll a/b-binding protein, and photosystem II stability/assembly factor HCF136 were down-regulated in response to salinity stress (Figure 7A). These three proteins are components of the light-harvesting complex of PS II in plants, and facilitate light absorption and transfer of the excitation energy to the reaction centers for charge separation (Takahashi et al., 2006). The electrons released from PSII are transferred into PSI via cytochrome complexes. PSI P700 apoprotein A1, a component of the PSI system in plants with thylakoid structural protein, and thylakoid luminal, which is a 16.5 kDa protein in the thylakoid, decreased in abundance in response to salinity stress. These results, combined with the physiological measurements of photosynthesis, implicated that 500 mM NaCl limited the light absorption and energy transfer of PS in seedling leaves and further affected photosynthesis (Figures 4, 8). Hence, large amounts of ATP are needed to provide energy for plant growth and cope with high salinity. ATP synthase is up-regulated under salt stress in the halophyte *Aeluropus lagopodes* (Sobhanian et al., 2010). In our proteomic analysis, chloroplast ATP synthase significantly increased after salt treatment, suggesting that ATPase could be involved in the tolerance of *T. Nitraria* to high salinity.

The Calvin cycle produces substrates for the synthesis of starch, sucrose, and other products. In plants, enzymes of the Calvin cycle respond differently to salt stress (Caruso et al., 2008). In this study, we identified six differentially expressed proteins that participated in the Calvin cycle. These proteins included ribulose bisphosphate carboxylase/oxygenase activase 1, ribulose-1,5-bisphosphate carboxylase/oxygenase large subunit, rubisco subunit binding-protein alpha subunit, carbonic anhydrase-like 2, phosphoglycerate kinase, and fructose-1,6-bisphosphatase. Four of these six proteins were down-regulated, whereas the remaining two were up-regulated (Figure 7A). Rubisco, which is located in the chloroplast stroma, is the key enzyme in the Calvin cycle. The abundance of ribulose-1,5-bisphosphate carboxylase/oxygenase large subunit (RuBisCO LSU) decreased under high salinity stress. Similar observations were detected in proteomic analysis on *K. candel* (Wang et al., 2013).

**Figure 9:**
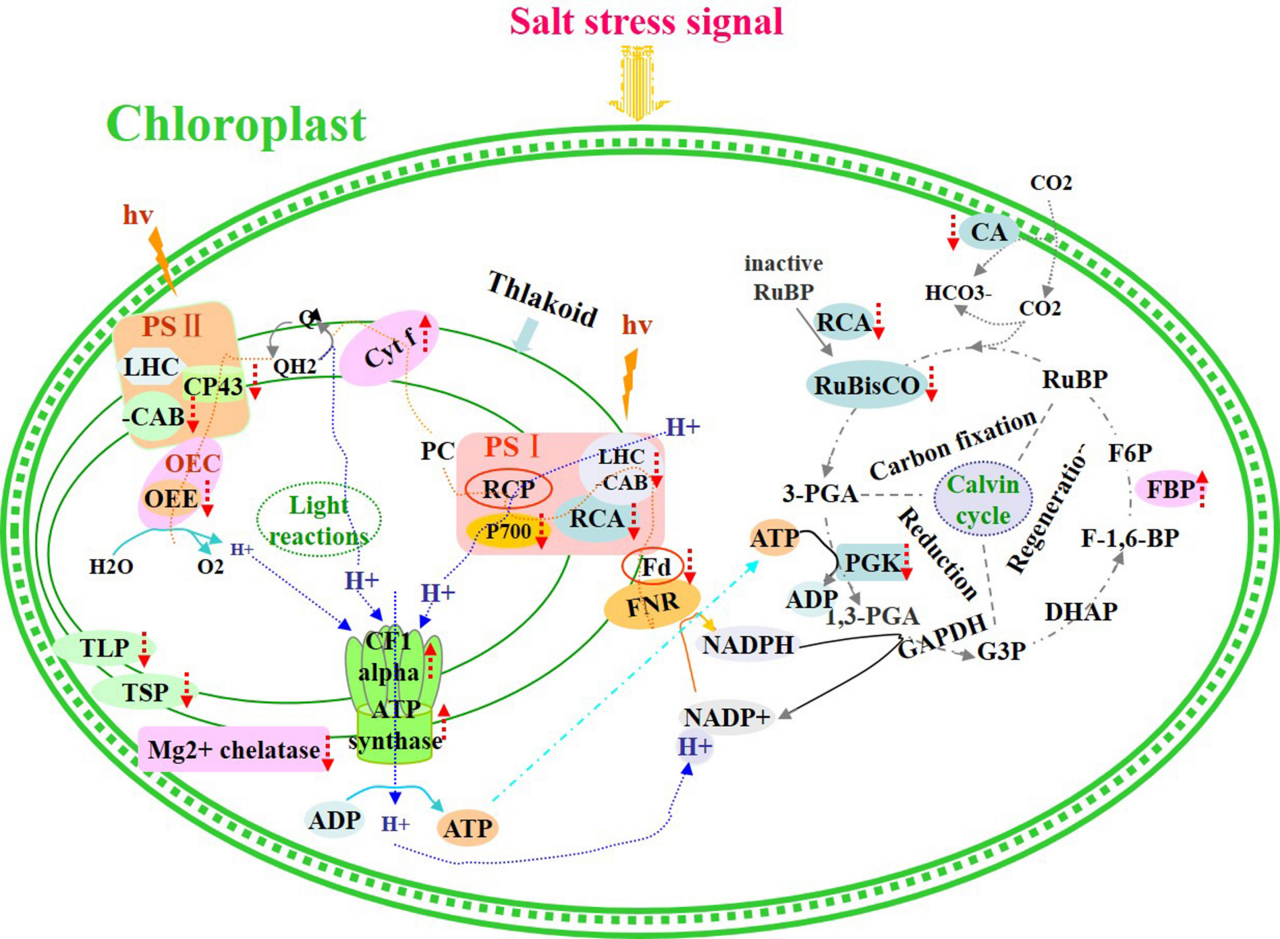
**Schematic presentation of salinity stress-responsive proteins involved in photosynthesis in *T. Nitraria* seedlings**.

Salinity stress ultimately inhibited photosynthesis in seedling leaves, and the proteins involved in this process are depicted in Figure 9. Our data obtained from plants under salt stress provide novel insights into the relationship between the effect of salinity stress on photosynthesis and protein expression regulation (abundance changes and breakdown) in seedling leaves.

### Proteins involved in antioxidative pathways are differentially expressed in *T. Nitraria* leaves

**Figure 10:**
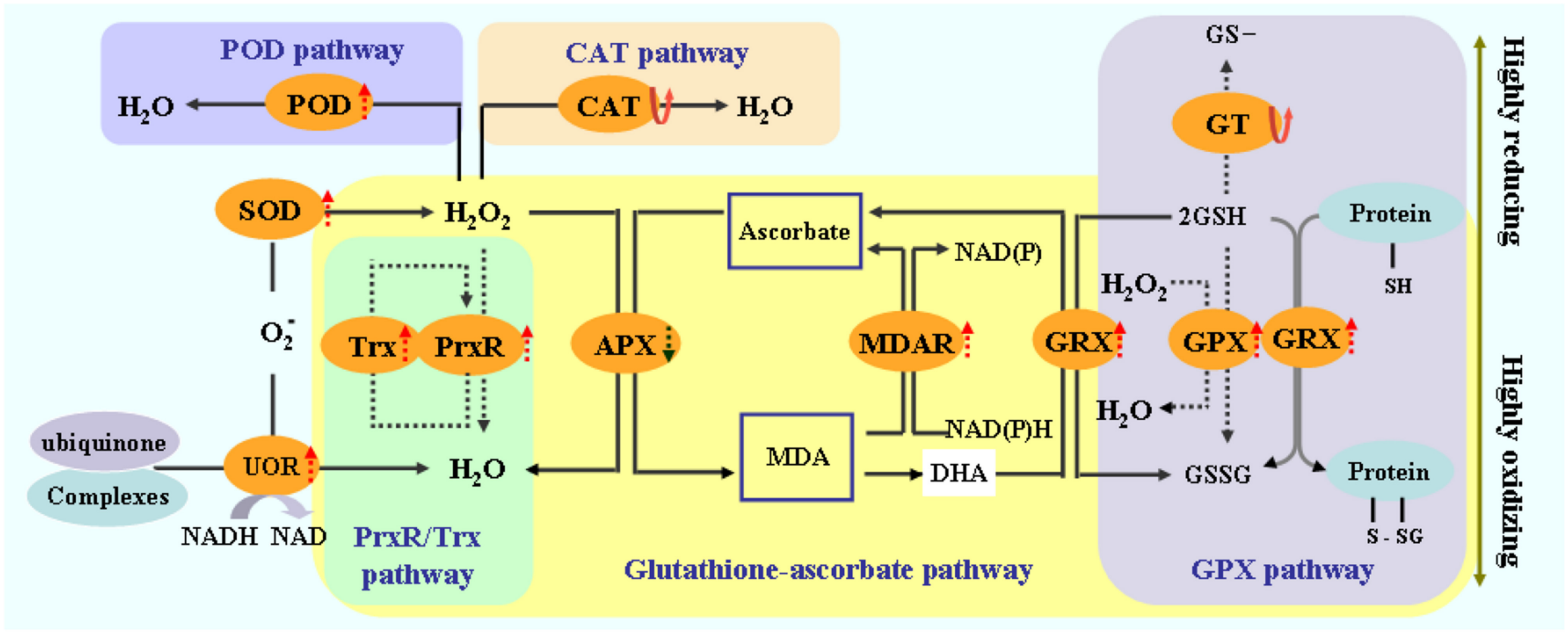
**Schematic presentation of salinity stress-responsive proteins involved in redox homeostasis in *T. Nitraria* seedling leaves**.

Plants protect themselves from injury via various biochemical and molecular adjustments, particularly via increased synthesis of protectants and antioxidants. In this study, the proteins that responded to high salinity included various antioxidants. A total of 15 identified proteins were involved in antioxidative reactions and belonged to redox homeostasis or stress/defense category (Figure 7B). Adequate regulation of the steady-state status and level of ROS in plants is necessary to avoid injury, as well as to perceive and transmit signals that are important in plant development and adaptation to the environment (Wan and Liu, 2008). Salinity stress causes a rapid increase in ROS and imbalance between ROS and the antioxidative defense system. Salinity stress also causes over-reduction of electron transport chain and various detoxification reactions, which are often accompanied with a rapid increase in reactive ROS, including superoxide radicals (•O^−^_2_), hydrogen peroxide (H_2_O_2_), and hydroxyl radicals (•OH); these ROS can perturb cellular redox homeostasis and damage many cellular components and structures (Cruz de Carvalho, 2008). We found that the 12 identified differentially expressed proteins were implicated in redox homeostasis. These proteins included putative L-ascorbate POD, MDAR, glutathione peroxidase, CAT, SOD [Mn], peroxidase precursor, oxygen-evolving enhancer protein 1, peroxiredoxin, thioredoxin peroxidase, glutathione transferase, ferredoxin–NADP reductase, and NADH-ubiquinone oxidoreductase 24 kDa subunit (UOR) (Figure 7B). These proteins are also involved in the CAT and POD pathways, glutathione–ascorbate cycle, peroxiredoxin/thioredoxin (PrxR/Trx) pathway, glutathione peroxidase (GPX), and SOD pathway (Figure 10). ROS scavenging systems were activated in the leaves to alleviate oxidative damages caused by salinity stress. Three enzymes, including L-ascorbate peroxidase (APX), GPX, and MDAR, showed similar expression patterns to X; these enzymes could remove H_2_O_2_ in the glutathione–ascorbate cycle (Figure 10). Glutathione transferase (GT) can also reduce H_2_O_2_ to the corresponding hydroxyl compounds and confer protection against oxidative membrane damage; the expression of this enzyme is strongly enhanced by biotic and abiotic stresses. NADH-UOR, peroxiredoxin, and thioredoxin peroxidase play important roles in the peroxiredoxin/thioredoxin-based redox pathway, which is included in the antioxidative defense system (Vieira Dos Santos and Rey, 2006). Thus, our results, in conjunction with those in the report of Zhang et al. (2011), demonstrated the up-regulation of these proteins. Such findings implied that the antioxidative defense system was provoked, and the ROS scavenging system was activated to alleviate oxidative damage by eliminating excess ROS for enhanced salinity tolerance.

### Plants may change protein metabolism to alleviate salinity stress

The proteins that were differentially expressed in *T. Nitraria* seedlings as a response to high salt concentrations included proteins involved in protein metabolism. These proteins also play a pivotal role in the tolerance of plants to salinity. Nine differentially expressed proteins involved in protein metabolism were categorized into three functional groups (Figure 7C). The first group consisted of five proteins involved in peptide biosynthesis. These five proteins were ribosomal protein L3A, glycyl-tRNAsynthetase 2, immunophilin/FKBP-type peptidyl-prolylcis-trans isomerase, aspartyl-tRNA synthetase, and peptidyl-prolylcis-trans isomerase. The second group comprised proteins important in folding and assembly. These proteins included chaperonin-60 kD, putative chloroplast heat shock protein 70-2, and chaperonin containing t-complex protein 1 epsilon subunit. These chaperonins have been well-studied, and they are known to be responsible for protein refolding and assembly (Wang et al., 2004). Figure 7C shows the up-regulated or down-regulated expression patterns of these proteins, which are responsible for protein folding and assembly, respectively. The third group contained 26S proteasome regulatory subunit 7, which is associated with protein degradation. The down-regulation of this protein may implicate that the plant decreased energy consumption to improve salt tolerance.

### Differentially expressed proteins are implicated in signal transduction

When plants are exposed to salinity stress, stress signals are perceived by a complex signaling network. In this study, 13 proteins involved in signal transduction and protein transport were differentially expressed in response to salinity stress (Figure 7D). Among these proteins, seven (G-box-binding factor, mitogen-activated protein kinase 4, phosphofructokinase, glycerol kinase, phosphoglycerate kinase, nucleotide binding protein, and ACC oxidase) were related to the signal transduction pathways. G-box-binding factor was up-regulated in the leaves. G-box-binding factor, a member of the GTP-binding protein family, participates in the small GTPase-mediated signal transduction pathway activated by external signals (Askari et al., 2006). Mitogen-activated protein kinase 4 (MAPK4) was initially down-regulated and then up-regulated under salinity stress. MAPK4 is a specific activator of the salt stress response (Kiegerl et al., 2000). ACC oxidase is a key enzyme that catalyzes the final step of ethylene synthesis with ACC as the substrate (Wang et al., 2002), thereby indicating a down-regulated pattern under salinity stress. This result inferred that ethylene may negatively regulate salt stress responses by down-regulating the expression of ACC oxidase in the leaves of *T. Nitraria*. Similar results were also observed by Dong et al. (2011), who found that *ACS7* exhibits a negative function in mediating salt stress responses.

### Models for the tolerance of *T. Nitraria* seedlings to salinity stress

Plant tolerance to salinity stress relies on diverse adaptive strategies, including regulation of morphology, physiology, biochemistry, and molecular genetics. In this study, we demonstrated that *T. Nitraria* tolerance and adaptation to salinity stress were associated with coordinated and ordered expression of proteins involved in photosynthesis, redox homeostasis, defense signaling pathways, signal transduction, and altered substrate metabolism. Our proteomic and physiological data demonstrated the following events that resulted in salinity tolerance or adaptation. This network consisted of several functional components, including imbalance between ROS production and scavenging, impaired photosynthesis, accelerated biosynthesis and reduced degradation of proteins, and an enhanced antioxidative defense system. These changes in metabolic reactions and redox balance eventually induced plants to a new homeostasis to adapt to salinity stresses. Investigations on protein networks involved in the high salt tolerance of *T. Nitraria* seedlings will contribute to develop strategies for improving salt tolerance in plants.

## Conclusion

In this study, we applied comparative proteomic approaches to obtain a comprehensive proteomic description of the salinity tolerance of the halophyte *T. Nitraria*. Several stress symptoms, including changes in morphology, photosynthesis capability, ultrastructure of thylakoids, and membrane integrity, occurred during the course of salinity treatment. Among the 71 salinity-responsive proteins identified in the leaves of *T. Nitraria* plants treated with 500 mM NaCl, the majority of proteins were involved in redox homeostasis, photosynthesis, signal transduction, energy, and carbohydrate and protein metabolism. Protein–protein interaction analysis implicated that many processes collectively functioned to re-establish cellular homeostasis under salt stress. These results generally describe the strategies of *T. Nitraria* seedlings to tolerate high salinity, and elucidate the functional networks involved in the tolerance. This research can reflect management of cellular activities in *T. Nitraria* under salinity stress, and provide a basis for further functional research on each protein in intracellular homeostasis.

### Conflict of interest statement

The authors declare that the research was conducted in the absence of any commercial or financial relationships that could be construed as a potential conflict of interest.
