# Supplementary material for: Physiological and proteomic analyses of leaves from the halophyte Tangut Nitraria reveals diverse response pathways critical for high salinity tolerance
Source: Front Plant Sci. 2015 Feb 10;6:30. doi: 10.3389/fpls.2015.00030 (PMC4322618; doi:10.3389/fpls.2015.00030)

Figure S1-S7

Figure S1. Maximum quantum efficiency of PSII photochemistry (*Fv/Fm*) in *Tangut Nitraria* seedling leaves under the treatment of 500 mM NaCl for 0, 1, 3, 5, 7 days. The values are presented as means ± standard error (SE).

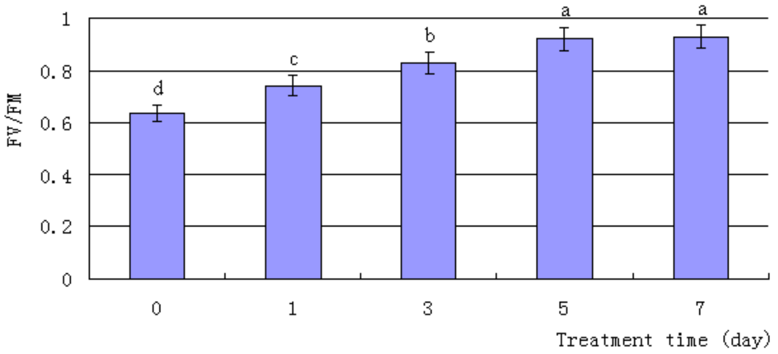

Figure S2. The effect of salinity on the ion content of *Tangut Nitraria* seedling leaves. The effects of increasing treatment time under salinity on K^+ (A)and Na^+ contents (B), and the K^+/Na^+ ratio (C) in the leaves of the treated plants. The values are presented as means ± standard error (SE).

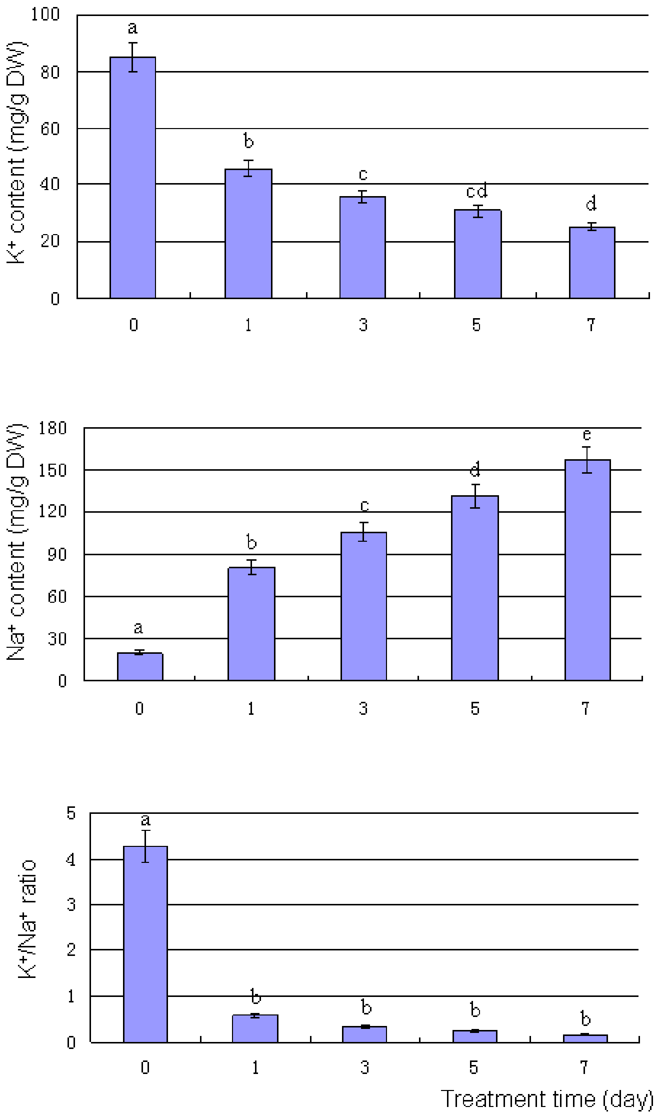

Figure S3. Effect of salinity on activities of CAT, POD, and SOD in *Tangut Nitraria* seedling leaves under the treatment of 500 mM NaCl for 0, 1, 3, 5, 7 days. The values are presented as means ± standard error (SE).

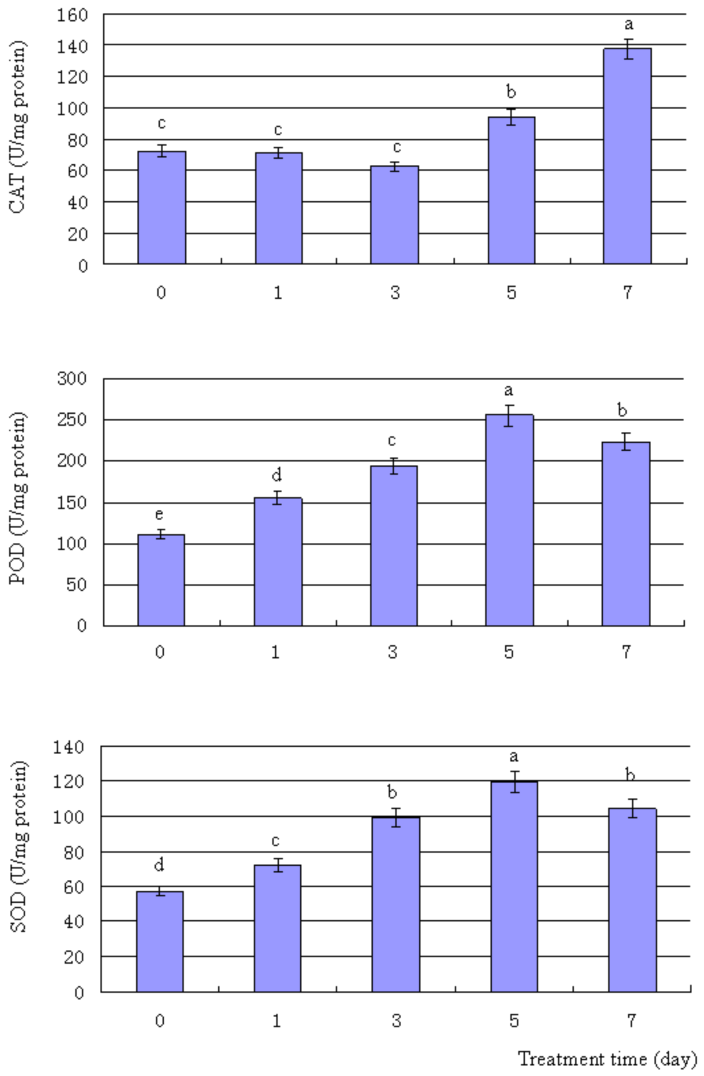

Figure S4. Contents of MDA and electrolyte leakage in *Tangut Nitraria* seedling leaves under the treatment of 500 mM NaCl for 0, 1, 3, 5, 7 days.

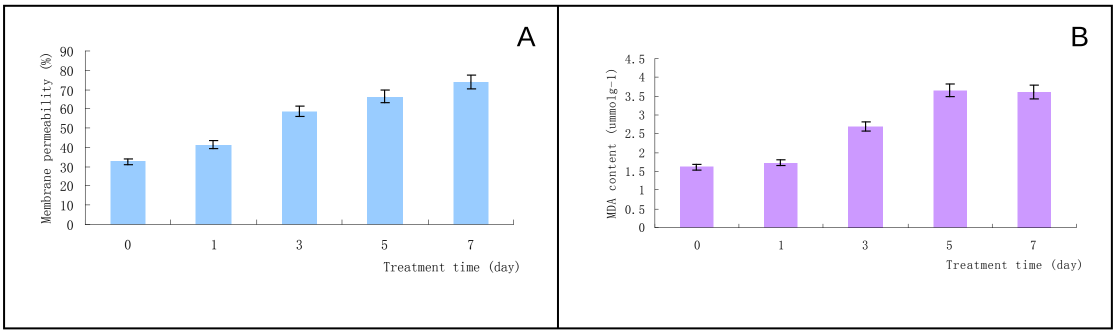

Figure S5. Experimental design of *Tangut Nitraria* response to salinity stress by iTRAQ-based proteomic approach.

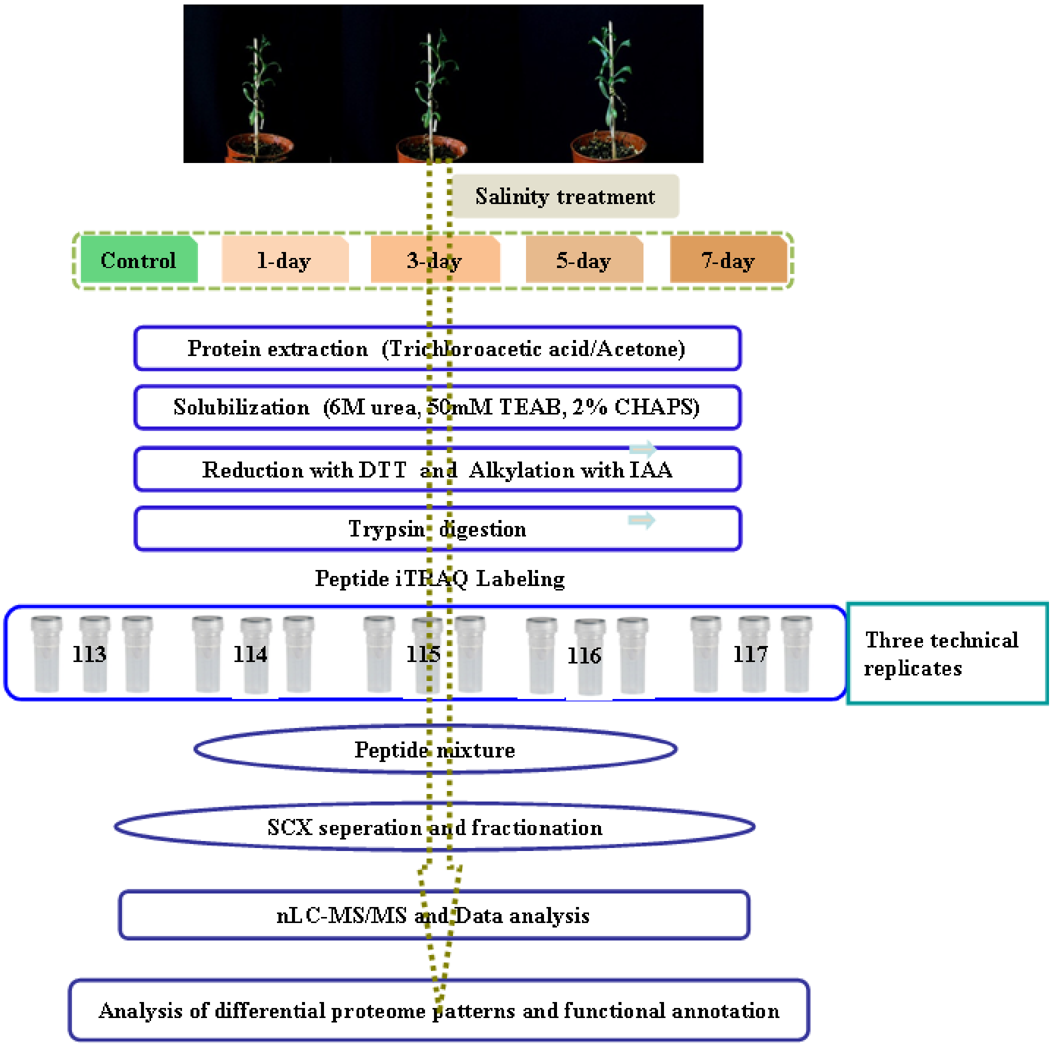

Figure S6. Hierarchical clustering of the changes in abundance of high salinity stress responsive proteins

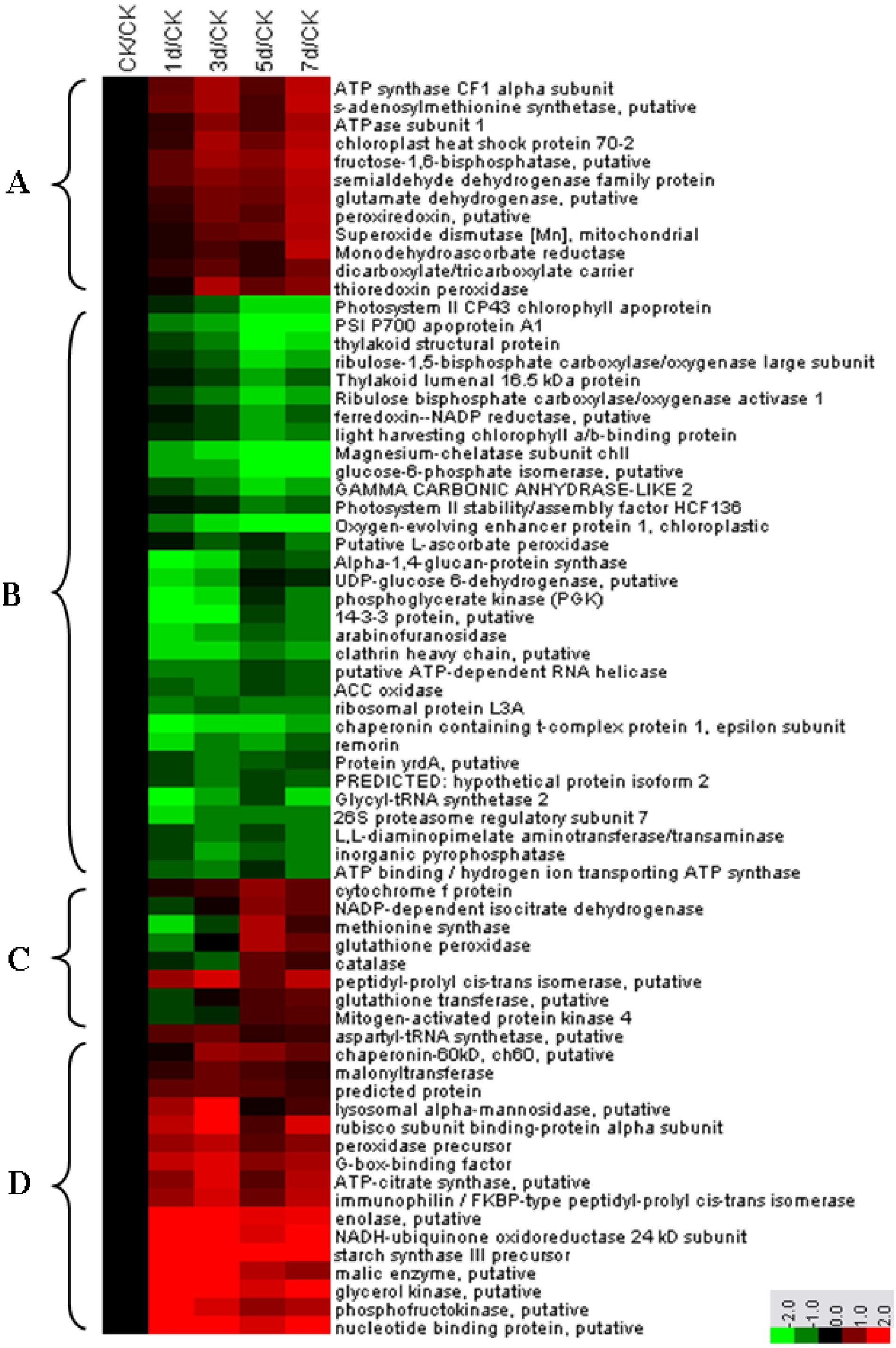

Figure S7. Functional classification of the differentially expressed proteins in leaves of *Tangut Nitraria* seedlings in response to salinity.

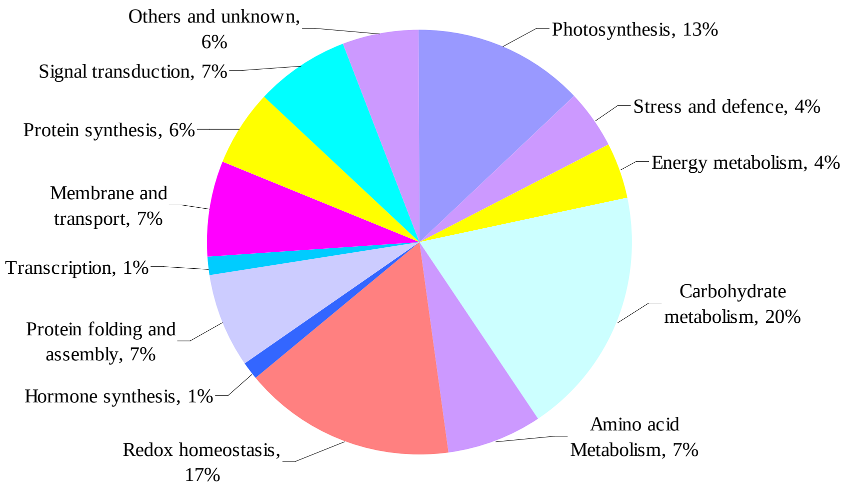

Supplement: Supplementary file 6 [file Image1.PDF]
